# Cell-in-cell phenomenon in urinary sediment: a case report

**DOI:** 10.11613/BM.2022.020801

**Published:** 2022-04-15

**Authors:** Carlos Martínez-Figueroa, Karen Cortés-Sarabia, José Antonio Tesser Poloni, Enrique Alejandro Molina-Avilez, Luis A. Palaoro, Amalia Vences-Velázquez

**Affiliations:** 1 InvesLab Clinical Laboratory, Iguala de la Independencia, Guerrero, Mexico; 2 Laboratory of Immunobiology and Molecular Diagnostics, Faculty of Chemical and Biological Sciences, Chilpancingo, Mexico; 3 Health School and Post Graduation Program in Food and Nutrition, Universidade do Vale do Rio dos Sinos, São Leopoldo, Brazil; 4 Controllab, Rio de Janeiro, Brazil; 5 Clinical Laboratory, Hospital Regional de Zona No. 2 "El Marqués", Querétaro, México; 6 Department of Clinical Biochemistry, University of Buenos Aires, Buenos Aires, Argentina

**Keywords:** urinary sediment, urinalysis, urinary tract infection, cell-in-cell

## Abstract

The internalization of apoptotic cells by non-phagocytic cells has been observed in different tissues and could be an important mechanism for the elimination of dying cells. Here, we describe a probable event of phagocytosis of apoptotic cells mediated by urothelial cells in urinary sediment. A 90-years-old male patient was admitted unconscious to the hospital, visible signs included: pale skin and dry mucous membranes, presumptively diagnosed as dehydration. Blood test revealed anaemia (haemoglobin 130 g/L) and hyperglycaemia (glucose 7.8 mmol/L), urinalysis showed a picture of urinary tract infection (leukocyturia and bacteriuria). The microscopic analysis of urinary sediment revealed the presence of urothelial cells and leukocytes internalized in urothelial cells. Anti-CD68 (membrane marker of macrophages) was tested by immunocytochemistry and a negative result was observed. Based on the findings phagocytosis of apoptotic cells mediated by urothelial cells was identified. This phenomenon can be observed in urinary sediment and should not be confused with a neoplastic process since it is a physiological event of cell elimination.

## Introduction

The cell-in-cell phenomenon is known as the internalization of cells by non-phagocytic cells such as epithelial or mesenchymal cells, these structures are commonly observed in benign or malignant conditions (nurse cells in thymus, Rosai-Dorfman syndrome, urothelial and renal cancer, *etc*.) (*1*, *2*). There are several variants of this phenomenon: i) Cannibalism - associated to the internalization of neoplastic cells by other neoplastic cell, ii) Entosis - internalization of one cell by a neighbouring cell (it can be benign or malignant), iii) Emperipolesis - the cells of the immune system are found within neoplastic or benign cells, and, iv) Phagocytosis of apoptotic cells by non-phagocytic cells - similar to efferocytosis (phagocytosis of apoptotic cells by macrophages) (*3*-*5*).

In recent years, the internalization of apoptotic cells by non-phagocytic cells has gained relevance because it can be observed in benign conditions or in non-neoplastic pathological processes (*5*, *6*). It is considered as a physiological mechanism for the elimination of apoptotic or necrotic cells and is carried out by various types of epithelial cells, among them; renal tubular epithelial cells and urothelial cells (*6*, *7*). This phenomenon has been evaluated using *in vitro* and *in vivo* models, by using tissue sections of kidney (*6*, *8*). The main objective of this case report is to present a rare cell-in-cell phenomenon found in fresh urine sediment preparation.

## Case description

**Figure 1:**
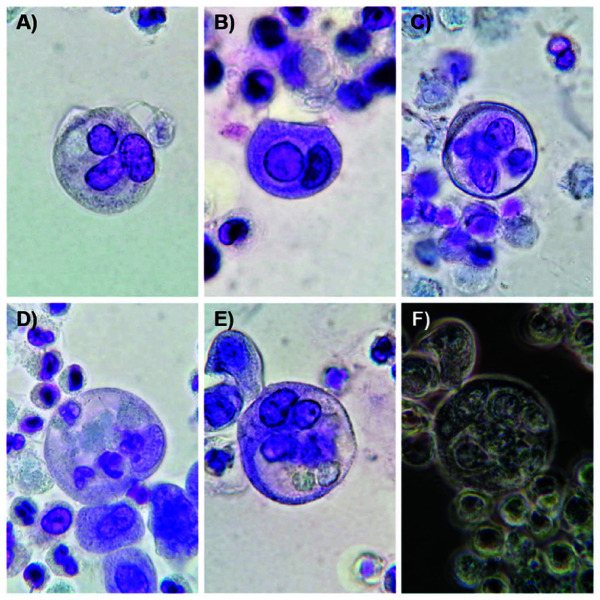
Observation of urothelial cells and leukocytes inside urothelial cell (cell-in-cell phenomenon) in urinary sediment preparation. Figures 1A-C - urothelial cells inside other urothelial cells. Figures 1D-E - leukocytes internalized by urothelial cell. Figures 1A-E: brightfield microscopy, Toluidine blue staining, original magnification 1000x. Figure 1F: leukocytes inside urothelial cell observed in the fresh and unstained urine sediment, phase contrast microscopy, original magnification 1000x.

**Figure 2:**
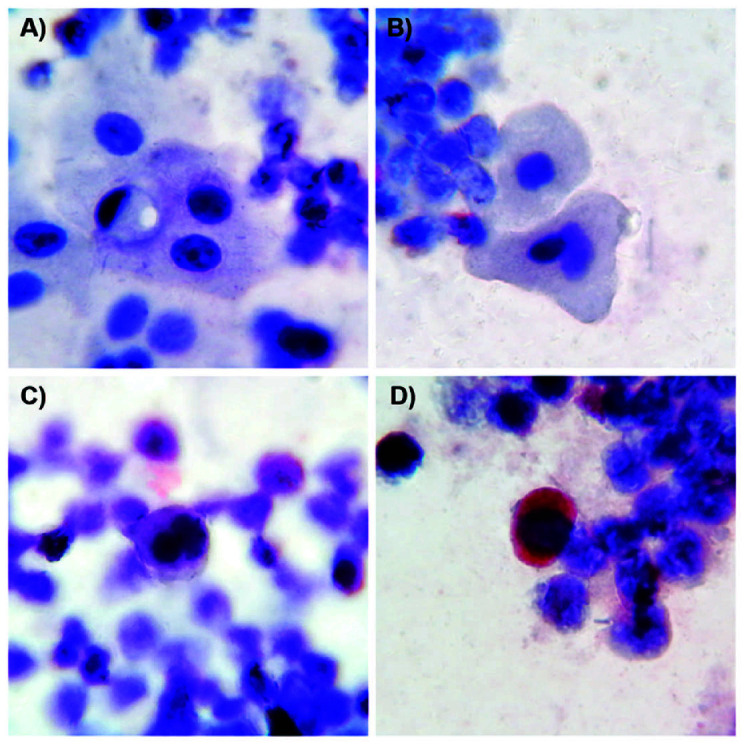
Anti-CD68 immunocytochemistry in urinary sediment preparation. Figures 2A-C - Representative images of negative staining of cell-in-cell structures derived from urinary sediment preparation. Figure 2D - Macrophages preparation was used as positive control. Brightfield microscopy, original magnification 1000x.

A 90-year-old male patient was admitted to the emergency department of the San Juan clinic in Iguala de la Independencia, Guerrero, Mexico. He was unconscious, with visible signs included dry mucous membranes and pale skin. His family reported a low fluid intake, and a presumptive diagnosis of dehydration was made. Upon admission, the patient’s vital signs were taken; blood pressure of 110/70 mmHg, heart rate of 75 beats *per* minute, temperature of 36 ˚C and oxygen saturation (SPO2) of 96%. Laboratory studies showed a slight picture of anaemia; haemoglobin 130 g/L (reference interval (RI) 135–175), erythrocyte count 4.7 x10^12^/L (RI 4.5–5.8), haematocrit 0.37 L/L (RI 0.42–0.52). Leukocyte count was 6.5 x10^9^/L (RI 4.5–11.0) and platelets 362 x10^9^/L (RI 150–450). The blood chemistry showed the following results: glucose 7.8 mmol/L (RI 3.9–6.1), urea 6.4 mmol/L (RI 2.5–6.5), blood urea nitrogen (BUN) 6.6 mmol/L (RI 2.5–7.1), creatinine 104 µmol/L (RI 53–106). Urinalysis was performed manually using Multistix 10 SG test strips (Siemens, Erlangen, Germany) and the sample was centrifuged at 450xg for 5 minutes, presenting the following results: relative density 1.025, pH 6.0, leukocyte esterase 2+ (approximately 125 leukocytes/µL) and protein 1+ (approximately 30 g/L). In the microscopic examination, moderate amount of urothelial and renal tubular epithelial cells (5–15 cells *per* high power field) were observed, and leukocytes > 100/high power field and bacteriuria were found. During the microscopic detailed review of the preparation, aqueous solution of toluidine blue (Tecnica Quimica, Mexico City, Mexico) at 1% was used as a contrast dye and the analyst noticed the presence of urothelial cells and leukocytes internalized in urothelial cells, for which a cell-in-cell phenomenon was suspected (Figure 1). To investigate if phagocytosis was carried out by macrophages, an anti-CD68 immunocytochemistry (glycoprotein expressed in the plasma membrane of macrophages) was performed. Mouse Anti-CD68 clone KP-1 (BIO SB, Santa Barbara, USA) was used as primary antibody, an anti-mouse IgG coupled to Alkaline Phosphatase (BIO SB, Santa Barbara, USA) was used as secondary antibody and Alk Magenta was used as chromogen. The immunostaining was negative (Figure 2), which correlates with a phagocytosis event performed by “non-professional phagocytic cells” associated with an inflammatory process of the urinary tract.

## Discussion

In this clinical case, it was observed evidence of cell-in-cell phenomenon in the urine sediment. To confirm that this phenomenon was not carried out by macrophages eliminating apoptotic cells, an immunocytochemistry against CD68 was performed and the result of this test was negative. For a long time, it was considered that the elimination of epithelial cells (from the kidneys and urothelium for example) and leukocytes in apoptosis, was carried out by macrophages. However, new evidence establishes that epithelial cells (*e.g.* renal tubular cell lines) play an important role in complementing the activity of macrophages by eliminating this type of cells (*6*).

In the literature, there is only one article that addresses this issue in urinary cytology, which establishes that cell phagocytosis is not an exclusive event of neoplastic processes, but is also observed in glomerular diseases. However, the authors could not explain its origin (*9*). This article is the first report of cell-in-cell phenomenon in urine cytology associated to non-malignant pathological processes. According to our findings, the cell-in-cell phenomenon described in this case provides evidence of phagocytosis performed by non-professional cells. Ichimura *et al*., observed that after kidney injury, survival renal tubular epithelial cells internalized the apoptotic bodies of remaining injured cells using *in vitro* (cell culture) and *in vivo* models (ischemia reperfusion injury in rat kidney) (*8*). Seeberg *et al*., studied five cell lines derived from kidney cells, and observed phagocytosis of dying cells (*6*). In a mouse model with urinary tract infection, Mintz *et al.*, described the presence of neutrophils within urothelial cells. Using an *in vitro* model, the same authors evaluated the internalization of neutrophils by bladder epithelial cells and similar results were observed (*7*). They concluded that this is a neutrophil elimination mechanism that contributes to the resolution of inflammation in which urothelial cells could play an important role (*7*). Accumulated evidence indicates that various epithelial cells including urothelial and renal tubular cells participate in the elimination of apoptotic cells (renal tubular cells, urothelial cells and leukocytes), which could explain the cell-in-cell phenomenon observed in this clinical case. The presence of these structures has not been described in urinary sediment because urine samples with inflammatory conditions such as urinary tract infection (UTI) contain a large number of cells that can obstruct visualization, and once the inflammation is eliminated, the structures are no more observed. They can appear during these transitory processes and their observation will depend on the experience of the analyst and the time in which the sample was collected. In conclusion, the observed cell-in-cell phenomenon observed in urinary sediment during the inflammatory processes could be non-professional phagocytosis by epithelial cells (in both, urothelial and renal tubular cells). Therefore, they do not indicate or predict neoplasia since it is a physiological event for the elimination of dying cells.

## References

[1] Krishna S Overholtzer M. Mechanisms and consequences of entosis. Cell Mol Life Sci. 2016;73:2379–86. 10.1007/s00018-016-2207-0 27048820 PMC4889469

[2] Mackay HL Muller PAJ. Biological relevance of cell-in-cell in cancers. Biochem Soc Trans. 2019;47:725–32. 10.1042/BST20180618 30850425 PMC6490704

[3] Mlynarczuk-Bialy I Dziuba I Sarnecka A Platos E Kowalczyk M Pels KK Entosis: From cell biology to clinical cancer pathology. Cancers (Basel). 2020;12:2481. 10.3390/cancers12092481 32883000 PMC7563411

[4] Gupta N Jadhav K Shah V. Emperipolesis, entosis and cell cannibalism: Demystifying the cloud. J Oral Maxillofac Pathol. 2017;21:92–8. 10.4103/0973-029X.203763 28479694 PMC5406827

[5] Arandjelovic S Ravichandran KS. Phagocytosis of apoptotic cells in homeostasis. Nat Immunol. 2015;16:907–17. 10.1038/ni.3253 26287597 PMC4826466

[6] Seeberg JC Loibl M Moser F Schwegler M Büttner-Herold M Daniel C Non-professional phagocytosis: a general feature of normal tissue cells. Sci Rep. 2019;9:11875. 10.1038/s41598-019-48370-3 31417141 PMC6695441

[7] Mintz D Salamon H Mintz M Rosenshine I Shpigel NY. Intraepithelial neutrophils in mammary, urinary and gall bladder infections. Vet Res. 2019;50:56. 10.1186/s13567-019-0676-5 31324217 PMC6642505

[8] Ichimura T Asseldonk EJPV Humphreys BD Gunaratnam L Duffield JS Bonventre JV. Kidney injury molecule-1 is a phosphatidylserine receptor that confers a phagocytic phenotype on epithelial cells. J Clin Invest. 2008;118:1657–68. 10.1172/JCI34487 18414680 PMC2293335

[9] Ohsaki H Haba R Matsunaga T Nakamura M Kiyomoto H Hirakawa E. “Cannibalism” (cell phagocytosis) does not differentiate reactive renal tubular cells from urothelial carcinoma cells. Cytopathology. 2009;20:224–30. 10.1111/j.1365-2303.2009.00655.x 19563449

